# Emotional climate: a bibliometric analysis of the psychological consequences of climate change

**DOI:** 10.3389/fpsyg.2025.1521771

**Published:** 2025-07-09

**Authors:** Yadgar Momenpour, Shahla Choobchian

**Affiliations:** Department of Agricultural Extension and Education, Faculty of Agriculture, Tarbiat Modares University (TMU), Tehran, Iran

**Keywords:** climate emotions, climate psychology, scientific mapping, co-word analysis, bibliographic coupling, Scopus

## Abstract

Research on climate change and its impact on individuals' emotions are receiving increasing attention. Therefore, it is crucial to evaluate the current state of this research and predict future trends to offer greater clarity for researchers and decision-makers. This study aims to analyze the co-authorship network, bibliographic coupling, and co-word analysis of scientific documents produced by researchers in the Scopus database from 2010 to 2024. This period is characterized by the emergence and conceptual consolidation of emotional responses to climate change in academic research, focusing on the psychological consequences of climate change. A total of 1,333 documents were chosen for analysis after completing the identification and screening processes. This research is applied and descriptive in terms of its scient metric approach and is conducted using co-occurrence word analysis and network analysis techniques. The collected data were analyzed using Excel, and VOS viewer software was utilized to create visual maps. The co-occurrence analysis of high-frequency terms highlighted emerging topics and critical issues related to climate change. The findings emphasize the connection between clusters related to climate change and climate justice with the core cluster of humanity and human emotions, highlighting the emerging nature of this research domain. The thematic clusters identified further emphasize the significance and novelty of climate-related emotions and the organization of related research. This review can serve as a roadmap for future research, planning, and decision-making in the area of climate change and its associated impacts.

## 1 Introduction

Climate change (CC) is already underway, primarily driven by rising global temperatures, which are altering the Earth's climate system. As a result, extreme weather events and anomalies, such as heatwaves, intense storms, floods, wildfires, and droughts, are becoming more frequent and severe. In addition to these immediate manifestations, gradual but persistent changes, including sea level rise and shifts in precipitation patterns, are expected to have profound and long-term impacts on ecosystems and human societies in the coming decades (Clayton, 2020; Coffey et al., 2021). While it is often believed that CC mainly impacts Polar Regions, evidenced by melting ice caps and the extinction of polar species (Born, 2019), evidence shows that it also significantly affects human wellbeing (Lammel, 2025). Heat exposure, the transmission of diseases through contaminated water and insect vectors, and malnutrition pose significant threats to physical health. Additionally, the immediate impacts of socio-natural disasters like forced migration and conflict are pressing concerns (Watts et al., 2019). The psychological impacts of CC, including emotional responses (e.g., eco-anxiety, climate grief) and mental health outcomes, are often understudied despite their significance for human wellbeing (Stanley et al., 2021). Certain groups, such as indigenous peoples, the elderly, children, and those with preexisting health conditions, are especially vulnerable to these impacts. Their heightened risk is due to increased exposure to climate-related threats, a lack of political or economic power, and various physiological factors (Clayton, 2020). Therefore, it can be concluded that CC presents a serious threat to human health, significantly affecting both physical and mental wellbeing. However, substantial evidence underscores this link (Watts et al., 2019). The psychological impacts of environmental crises are diverse (Coffey et al., 2021). Studies indicate a rise in post-traumatic stress disorder (PTSD), depression, anxiety, substance abuse, and even domestic violence following experiences with storms (Morganstein and Ursano, 2020). Furthermore, extensive research confirms that concerns about CC and environmental issues undermine mental health and wellbeing, contributing to adverse emotional responses (IPCC, 2022; Teo et al., 2024).

The impacts of CC are often more pronounced for individuals who have experienced significant trauma, and their ability to cope is closely tied to social support systems and resilience (Clayton, 2020). Climate-related disaster exacerbates these challenges by indirectly affecting physical and social infrastructure, leading to disruptions in essential services such as education, healthcare, the economy, and transportation. These disruptions further increase stress levels among individuals and pose serious threats to the mental health of vulnerable populations (Clayton, 2020). Maibach et al. (2010) highlight that a stable climate is the most essential factor in determining human health. In support of this, Clayton et al. (2017) highlight how CC contributes to mental health issues by inducing emotional distress. Many individuals report feeling profound grief, loss, and despair related to the impacts of CC. This sentiment extends beyond personal feelings, as people worry deeply about their futures, the futures of their children, and those of upcoming generations. As global awareness of climate issues continues to rise, it has become increasingly important to understand the anxiety that often accompanies this crisis. Research shows that feelings of anxiety regarding the state of the planet are widespread. This is evidenced by studies conducted across various regions, including Europe (Heeren et al., 2022), America (Ballew et al., 2019), Africa (Mahl et al., 2020), and China (Wu, 2021).

News reports have increasingly focused not only on environmental degradation but also on its psychological effects on individuals, such as anxiety, grief, and emotional fatigue (Coffey et al., 2021). In the context of globalization and expanding media networks, information about environmental disasters, regardless of geographic distance, has become more accessible, allowing people to emotionally engage with events they have not experienced firsthand. This mediated exposure fosters emotional responses such as climate anxiety, ecological grief, and solastalgia, which are increasingly recognized in the literature as central to the affective dimension of CC (Clayton, 2020). By emphasizing the role of indirect and vicarious encounters in shaping these emotions, this discussion contributes to an expanded understanding of how climate emotions are not only rooted in personal experience but also socially and globally constructed. While the physical health impacts of CC, such as heat-related illnesses and disease transmission, have been extensively studied (Berry et al., 2018), research on its psychological consequences is gaining increasing attention. CC triggers a range of psychological responses, including immediate distress caused by exposure to natural disasters like storms and wildfires, as well as longer-term effects such as eco-anxiety, climate grief, and diminished mental wellbeing (Clayton, 2020; Palinkas and Wong, 2019). These psychological impacts are often compounded by indirect stressors, such as displacement, economic instability, and disruptions to social infrastructure, which further exacerbate mental health challenges (Palinkas and Wong, 2019). This growing focus on the psychological dimensions of CC underscores the need for a comprehensive understanding of its effects on human mental health and wellbeing.

Berry et al. (2018) propose analyzing the connection between CC and mental health through a framework that considers interacting distant, intermediate, and immediate processes. Similarly, Palinkas and Wong (2019) and Doherty and Clayton (2011) categorize the mental health impacts of CC into acute events (e.g., storms), sub-acute events (e.g., droughts), and existential threats (e.g., sea level rise), or direct, psychosocial, and indirect effects, respectively. These frameworks inform the thematic scope of this study.

In recent decades, the field of environmental psychology has introduced several new concepts, yet their precise definitions and practical applications remain underdeveloped. Increasingly, researchers are focusing on the potential impacts of CC on mental health, particularly concerning emotional responses such as heightened anxiety (Coffey et al., 2021). Negative emotions arising from CC and environmental crises, such as sadness, anxieties, and ecological guilt, are linked to a variety of individual reactions. Research indicates that higher levels of ecological guilt, often triggered by awareness of environmental degradation, including CC, are associated with pro-environmental behaviors or intentions to act (Moore and Yang, 2020). In the context of this study, we focus on how such guilt may relate specifically to CC-related actions. However, it is important to note that intentions do not always translate into actual behavior (Ajzen, 2020; Yi, 2021). Additionally, negative emotions related to CC can lead to feelings of hopelessness, which may manifest as denial and avoidance of environmental issues rather than prompt proactive responses (Ogunbode et al., 2021). Several factors increase the likelihood of experiencing heightened ecological anxiety, particularly in the context of CC. These include a stronger connection with nature (Gago et al., 2024) and a younger age, with studies indicating that youth are more susceptible to climate-related anxiety (Berry et al., 2018; Boluda-Verdu et al., 2022). However, ecological anxiety may also arise from broader environmental concerns, such as pollution or habitat loss, which are addressed in related literature.

CC affects individuals differently, but due to the interconnectedness of environmental issues in our global ecosystem and the existing evidence, it remains unclear whether the psychological effects of CC are unique compared to other environmental stressors. Research on the mental health outcomes related to concerns about CC has been limited so far. Despite the growing importance of studies in this area, there is still a limited understanding of the psychosocial experiences of mental wellbeing (Hogg et al., 2021). Existing measures of climate-related mental health and wellbeing tend to focus on negative emotions, such as worry and anxiety that people may experience when thinking about CC and other environmental issues (Stanley et al., 2021; Coffey et al., 2021; Léger-Goodes et al., 2022). CC affects individuals unevenly, with psychological impacts like eco-anxiety and climate grief often intertwined with broader environmental stressors, such as pollution or biodiversity loss. This study focuses on the psychological effects of CC, as evidenced by terms like “climate anxiety” and “climate grief” in the literature, while acknowledging that other environmental stressors may also contribute to similar emotional responses. Further research is needed to differentiate the unique psychological impacts of CC from those of broader environmental concerns (Hickman, 2020). Overall, this research aims to trace the evolution of studies on the psychological impacts of CC and to identify new and emerging areas for future research in this field. Therefore, bibliometric analysis is an effective and efficient method to achieve this goal.

This study focuses on the psychological impacts of CC, which are defined as the emotional, cognitive, and behavioral responses to climate-related stressors. These responses include eco-anxiety, climate grief, mental health disorders, and outcomes related to wellbeing such as resilience and coping strategies. The term “climate emotions” emphasizes specific affective responses like anxiety and sorrow within this broader framework. “Mental health” and “mental wellbeing” refer to both clinical and non-clinical psychological states that are influenced by CC. The goal of this study is to connect public health, environmental psychology, and affect theory within the context of climate discourse.

## 2 Methodology

### 2.1 Research design

Bibliometric analysis was chosen for this study due to the large volume of literature (over 2,000 initial articles), the interdisciplinary nature of the field, and our objective to provide a macro-level snapshot of research trends from 2010 to 2024. The time frame for this bibliometric analysis was set from 2010 to 2024. This decision was based on the observation that scholarly interest in the emotional and psychological dimensions of CC, such as eco-anxiety, climate grief, and climate-related emotional responses, began to gain conceptual clarity and publication momentum after 2010. Earlier literature was either sparse or lacked consistent terminology. Moreover, this period aligns with growing global attention to CC following the 2009 Copenhagen COP summit, which likely catalyzed the emergence of climate emotion discourse in the academic literature. As such, this time frame ensures both thematic relevance and methodological rigor. Using a longitudinal bibliometric approach, this research examines the psychological consequences of CC, based on data extracted from a premier academic database. Unlike traditional literature review approaches (such as integrative, narrative, scoping, and systematic reviews) that complement findings by providing deeper insights into specific subtopics or study quality, this method emphasizes describing the volume and scope of scientific research on a specific topic using various publication criteria. However, this analysis lacks descriptions of individual studies, assessments of their quality, insights into the effectiveness of interventions, or a synthesis of the overall strength of evidence in the research area. Instead, it offers a broad overview of a specific topic and the potential future research directions within a defined time frame.

### 2.2 Bibliometric analysis technique

Bibliometrics is a quantitative research method that utilizes bibliographic data, specifically publications and citations, to evaluate and map scholarly output (Broadus, 1987). Over the past decade, this approach has gained prominence in the publishing industry due to the rapid growth of scientific research, averaging 1,021 publications per year (Donthu et al., 2021). Bibliometric analysis offers a structured means to assess the performance, development, and future trends of research by examining patterns in authorship, keywords, publication sources, and geographic contributions (Fauzi et al., 2023; Xu et al., 2024). It encompasses both performance analysis, which measures research impact, and science mapping, which visualizes relationships among research components (Donthu et al., 2021). A key strength of bibliometrics lies in its ability to transform extensive, abstract scientific literature into a manageable and insightful format, enabling a deep understanding of a field's intellectual and thematic evolution (Fauzi et al., 2023; Zakaria et al., 2023). Among its techniques, co-citation analysis identifies conceptual linkages between frequently co-cited documents, revealing the intellectual structure of a field (Saleem et al., 2024; Andrews, 2003), while co-word analysis investigates the thematic relationships between frequently co-occurring keywords, shedding light on emerging topics and future directions (Liu et al., 2012; Saleem et al., 2024). The suitability of bibliometric methods depends on the scale of existing literature; larger datasets (e.g., over 500 publications) are best served by bibliometric analysis due to the impracticality of manual reviews (Donthu et al., 2021). Given that our initial search retrieved over 2,000 documents on the psychological impacts of CC in Scopus from 2010 to 2024, bibliometric analysis is a fitting methodological choice. Our study employs co-citation analysis to trace foundational literature, bibliographic coupling to examine current research alignments, and co-word analysis to uncover thematic developments and future directions. This integrated approach aims to provide a comprehensive, multi-dimensional understanding of how research on the psychological effects of CC has evolved, highlighting its interdisciplinary nature and forecasting its trajectory. While bibliometric analysis is a powerful tool for mapping the intellectual and thematic structure of a research field, we acknowledge that it does not capture the nuanced content or quality of individual studies. Our intention was not to assess causality or the effectiveness of specific interventions but rather to provide a macro-level overview of research trends, thematic evolution, and knowledge gaps in the literature on climate-related psychological impacts. This approach serves as a foundation upon which more targeted systematic reviews or meta-analyses could be constructed in future work.

### 2.3 Data collection, screening, and preparation

Among various databases, Scopus was selected for this study because it provides extensive access to a wide range of research-focused databases. Scopus is renowned for its depth and comprehensive coverage of high-quality scientific articles and is recognized as the most accepted and widely used database for bibliometric analysis (Van Nunen et al., 2018). Additionally, it is recommended in bibliometric research to utilize a single database to minimize the need for data integration and to reduce errors that may arise from using multiple databases (Donthu et al., 2021).

The study focused on the “psychological impacts of CC” in English-language journal articles published after 2010, using Scopus as the primary database. After conducting the initial search on Scopus, the results were downloaded in sets of 25 and combined into a master spreadsheet. Three members of the research team, including the first author and two research assistants, reviewed each article by examining the titles, abstracts, and keywords to determine if they met the inclusion criteria for the study.

For this research, we examined articles related to the psychological impacts of CC in the Scopus database. We searched for terms such as “Eco-anxiety,” “Climate trauma,” “Climate justice,” “Climate mental health,” “Climate grief,” and “Climate anxiety” within the 2010–2024 timeframe. Our focus was on the database's subject section, which includes titles, abstracts, and keywords. We also conducted co-word analysis on keywords to ensure that similar terms representing the same concept (e.g., Environmental anxiety and Eco-anxiety) were included in the analysis process. This was achieved by using the asterisk (^*^) during the search to identify and combine words or phrases that have the same meaning but may differ in spelling.

**Figure 1:**
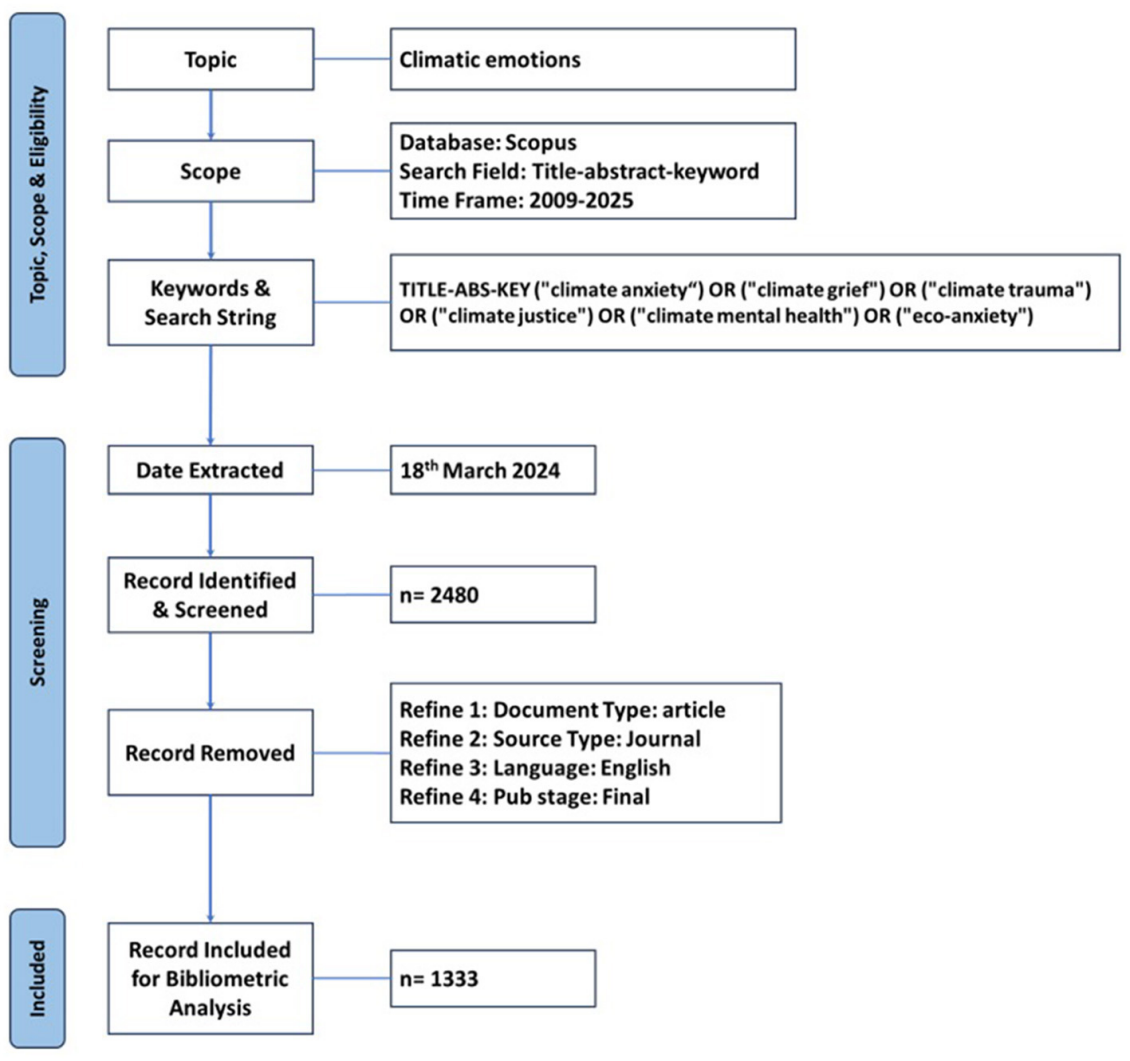
PRISMA flowchart (Page et al., 2021).

The search yielded 2,480 articles published between 2010 and March 18, 2024. We applied a filter to limit the documents to English-language research articles published in journals within the subject areas of Medicine, Social Sciences, Psychology, Environmental Science, Earth and Planetary Sciences, Agricultural and Biological Sciences, and Health Professions. We then reviewed the titles and keywords of each article. Articles that did not contain the searched terms in their titles or keywords were excluded from the final sample. After applying these limitations, 1,333 articles were selected and analyzed. In other words, the sample size for this study consists of 1,333 articles indexed in the Scopus database between 2010 and 2024 (Figure 1).

### 2.4 Analysis and reporting

To analyze the data and explore the relationships among various variables in this research, Social Network Analysis was employed. Social Network Analysis focuses on understanding the relationships and interactions between variables rather than merely examining the data and variables themselves (Borgatti et al., 2009). This approach emphasizes the overall connections among variables (Malacina and Teplov, 2022). VOSviewer, a well-known tool for social network analysis, was chosen for its powerful graphical features, which improve the clarity and visualization of bibliometric data for researchers and readers alike (Van Eck and Waltman, 2010). Performance analysis was conducted using VOSviewer, which provided essential insights into the dataset and highlighted summaries of leading countries and journals in the field. For document-level analysis, including co-citation analysis, the content of each document was examined through titles, abstracts, and keywords from research articles. The authors then examined the content of each cluster and reached a consensus on the main theme of each. Data on all authors who have contributed to the primary literature was compiled through internet searches in order to conduct an author-level citation analysis. For newer authors, their research areas were determined by reviewing their departmental profiles at their current institutions, when available.

### 2.5 Validation of results

To ensure the accuracy, reliability, and scientific rigor of the bibliometric findings, this study adopted a comprehensive validation framework based on four key criteria: validity, confirmability, reliability, and transferability, as recommended by Saleem et al. (2024) and Lim and Kumar (2024). Validity was established through methodological triangulation. Specifically, three complementary techniques were employed to analyze the same dataset: (1) co-word analysis to identify emerging themes and future research trends; (2) author co-citation analysis to uncover the intellectual and theoretical structure of the field; and (3) bibliographic coupling to examine contemporary research trajectories. The simultaneous application of these three techniques on a unified dataset (i.e., documents indexed in the Scopus database) enhanced the internal validity of the findings and facilitated a multi-level exploration of knowledge structures. The decision to adopt triangulation over complementarity was grounded in the study's objective, which was to investigate the intellectual development and trajectory of research on the psychological impacts of CC through temporal dimensions (past, present, and future). Unlike complementarity, which is more suited to multi-source or mixed-method designs, triangulation in this context enabled a comprehensive and integrated analysis of a single large-scale dataset.

Confirmability was ensured through transparent documentation of all research procedures, including the design of the search formula, data filtering criteria, keyword normalization, and analytical methods applied using Excel and VOSviewer software. Each analytical decision and step was reported in a reproducible manner, enhancing the auditability of the research. Reliability was addressed by comparing the study's results with findings from similar prior research in the field (e.g., Berry et al., 2018; Clayton, 2020; Coffey et al., 2021). The consistency in thematic clusters, the recurrence of high-frequency keywords, and alignment in research patterns indicate the robustness and stability of the current findings over time. Although the study was centered on a single core research question—identifying the structure and evolution of climate-related psychological research—it was inherently multidimensional in scope. The analytical strategy purposefully incorporated temporal and thematic layers to enrich the interpretive depth. Methodological triangulation served not only to provide a broader analytical lens but also to enhance the explanatory power of the results. Transferability was achieved through the identification of distinct thematic clusters and sub-domains, allowing for meaningful generalizations across related interdisciplinary fields, such as environmental psychology, public health, and climate policy. These well-defined knowledge areas support the broader applicability of the study's insights to both academic and policy-related contexts.

In summary, the adoption of this robust validation framework significantly strengthens the credibility of the research findings and ensures their utility as a reliable source for scholars, practitioners, and policymakers interested in the psychological consequences of CC.

## 3 Results

**Figure 2:**
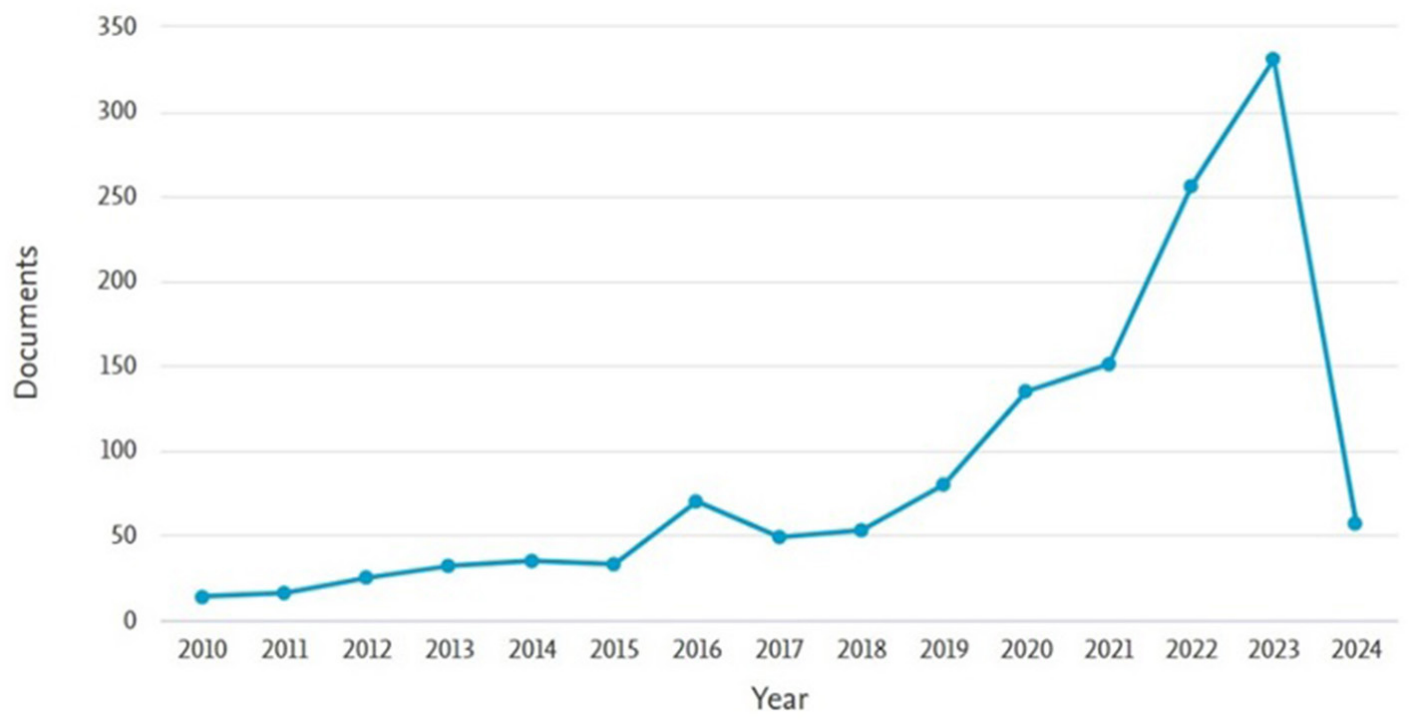
The trend of article publications over time.

The Scopus database provided a total of 2,480 citations for the selected articles (*N* = 1,333), excluding self-citations, resulting in an overall citation count of 22,198. The average number of citations per article was calculated to be 16.65, and the H-index was determined to be 67. A growing interest in research on the psychological impacts of CC was reflected by the substantial number of articles. Significant recognition in this field was not achieved until 2010, despite the publication of relevant journals over recent decades. These findings suggest that increased research on the psychological impacts of CC is likely. Trends in publication and citation data from 2010 to March 22, 2024, are illustrated in Figure 2.

### 3.1 Performance analysis

Table 1 presents initial information about the bibliometric data. The results show that the final sample consists of 1,333 articles published between 2010 and 2024 on the psychological impacts of CC. These articles were widely disseminated and sourced from 2,480 distinct publications. A total of 840 researchers contributed to these articles, with 589 being sole authors, while the majority was written by multiple authors. The collaboration index, calculated as the ratio of total authors to multi-authored papers, was 2.10.

**Table 1:** Publication and citation-related metrics.

**General description**	**Results**
Timespan	2010–2024
Sources (Journals)	148
Documents	2,480
Average citations per document	16.65
Average citations per year per document	1,585.57
**Document contents**	
Keywords plus	5,129
Author's keywords	3,145
**Authors**	
Authors	2,800
Authors of single-authored documents	549
Authors of multi-authored documents	784
**Authors collaboration**	
Authors pre citations	0.13
Authors per document	2.10

The average number of citations per document was 16.65, resulting in an annual average of 1,585.57 citations. The selected articles contained a total of 5,129 keywords provided by the authors.

### 3.2 Publication trends of articles

Figure 2 illustrates the annual trend in scientific publications related to the psychological effects of CC. Until 2019, there was limited growth in scientific articles; however, there has been a considerable increase in publications since that year. In general, the overall annual growth rate during the entire period was 37.85%. The growth rate for the first decade (2010 to 2020) was 13%, whereas, between 2020 and 2023, it soared to 133.33%. Additionally, the number of published articles has shown a significant upward trend, with the peak occurring in 2023, when 331 articles (24.75%) were released. In the last 3 years, from 2021 to 2024, a total of 795 articles were published, making up 59.46% of all articles published during this time. This indicates that researchers are increasingly focusing on this area of study, and it is expected that this upward trend will continue in the coming years. It is important to note that not all accepted papers had been published by the time of this study, and many research projects are still under revision by their authors. Moreover, since we are still early in the year, the number of articles published in 2024 is currently lower than that of previous years.

### 3.3 Prominent authors in this field

**Figure 3:**
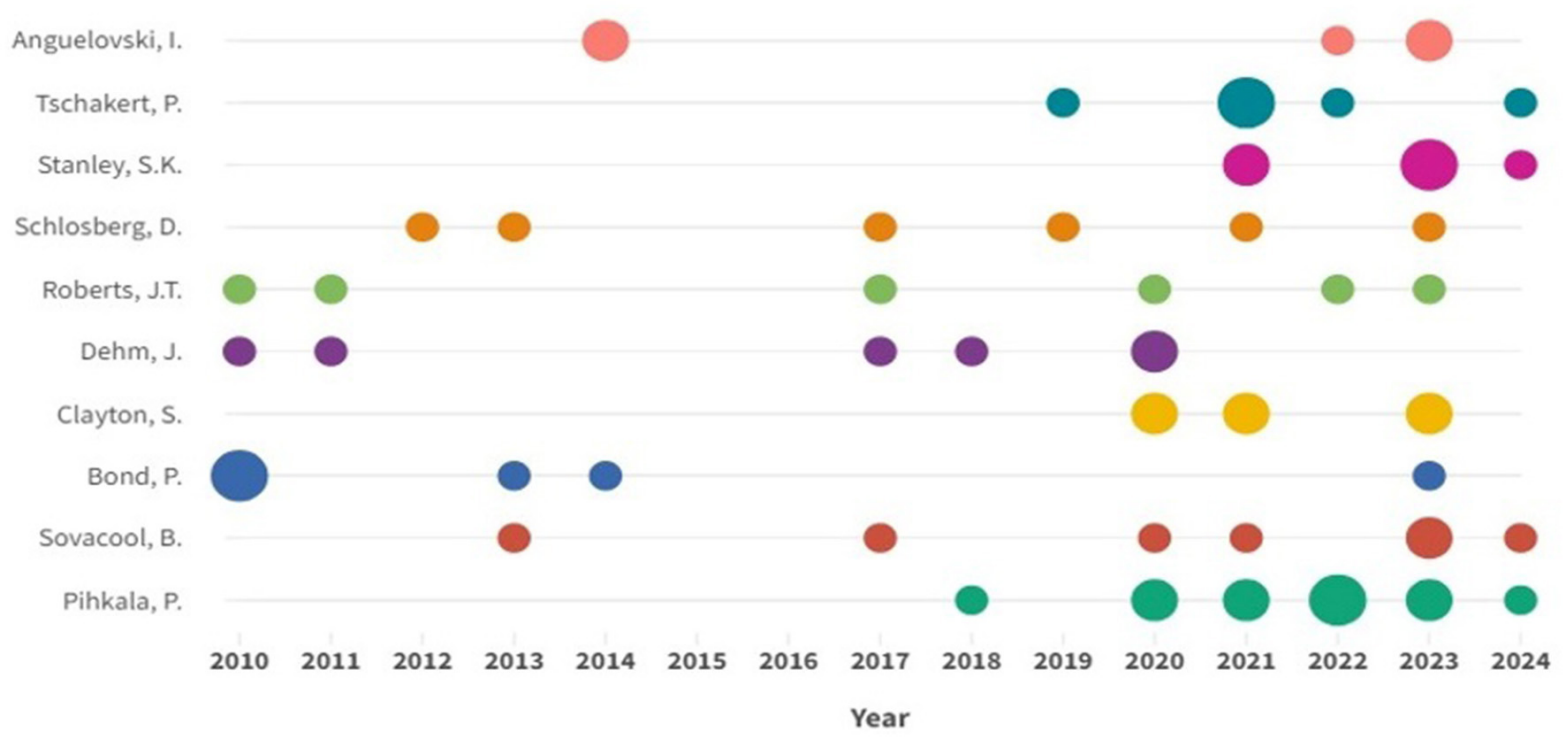
The productions of the top authors over time. The size of the circles represents the number of articles published annually.

Table 2 highlights the 10 most productive authors based on the number of articles published and the number of local and global citations. Pihkala, P. from Finland is the most prolific author, with 12 research articles (Figure 3). Sovacool, B. ranks first in total citations, with 35,767 citations. Clayton, S. has the largest influence in this field due to having the most local citations. Among the authors, Pihkala, P. and Sovacool, B. K. are the most productive, with 12 and 7 articles, respectively. Clayton, S. and Schlosberg, D. are also prominent, with 1,145 and 1,047 citations, respectively. These four authors stand out as leading figures in both the number of publications and citations, underscoring their significant contributions to advancing research on the psychological impacts of CC. Moreover, the study reveals that while the United States has the highest overall number of authors, the majority of the top ten authors are from Australia, with four representatives in the group.

**Table 2:** Most prominent authors.

**Authors**	**Institution**	**Faculty/Department**	**Articles**	**Global citations**	Local citations^*^	**h-index**
Pihkala, P.	University of Helsinki	Helsingin Yliopisto	29	3,629	697	23
Sovacool, B.	Aarhus Universitet	Energy Policy at the Science Policy Research Unit	597	35,767	684	96
Bond, P.	University of Johannesburg	Sociology, Center for Social Change	186	2,089	262	25
Clayton, S.	Info The College of Wooster	Psychology; Liaison to the Environmental Communication and Action Pathway	104	5,784	1,145	38
Dehm, J.	La Trobe University	College of Arts, Social Sciences and Commerce	24	138	44	7
Roberts, J. T.	Brown University	Environmental Studies and Sociology	97	7,071	424	37
Schlosberg, D.	University of Sydney	Environmental Politics	57	6,675	1,047	27
Stanley, S.	The Australian National University	Research School of Psychology	32	521	267	10
Tschakert, P.	Curtin University	Faculty of Humanities	79	4,842	148	32
Anguelovski, I.	Universitat Autònoma de Barcelona	The Gender, Diversity, and Wellbeing Committee at ICTA	122	6,406	340	42

^*^Local citations as citations received from within the dataset used for analysis, in contrast to “global citations,” which refer to total citations indexed in the broader database.

**Figure 4:**
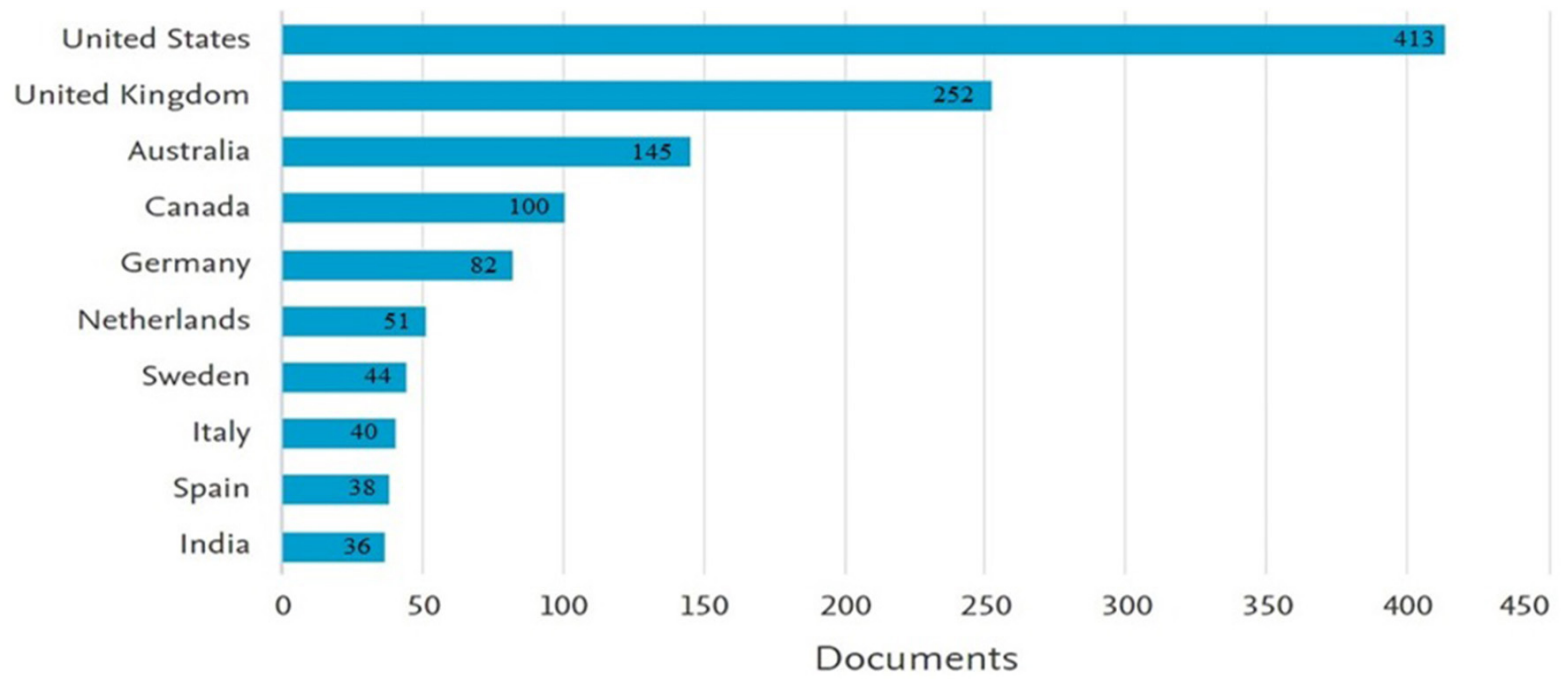
The top 10 countries in producing articles on the psychological impacts of climate change.

In the following descriptive analysis, the countries that produced articles on climate emotions were examined. Figure 4 displays the top 10 countries worldwide along with the total number of articles produced on climate emotions over 12 years, broken down by country. As shown in Figure 4, the United States and the United Kingdom are the top two countries, with 413 and 252 articles, respectively. Consistent with most research fields, the United States ranks highest. Following them are Australia, Canada, and Germany. According to Figure 4, India ranks 10th with 36 articles and is the only Asian country in this ranking.

### 3.4 Leading journals in this field

Table 3 presents the five most prominent journals in the field of CC concerns. According to the findings, *Climatic Change, Global Environmental Change*, and *Sustainability Switzerland* are the most prolific journals in this area, with 28, 23, and 22 articles published, respectively. In terms of citations, *Geoforum, Climatic Change*, and *Global Environmental Change* lead the field, with 675, 617, and 617 citations, respectively. Although *Geoforum* has published fewer articles, it ranks first in citations by a significant margin, highlighting its influence and leadership in this research area. Beyond publication numbers and citations, the h-index (these h-index values represent the overall journal metrics and are not specific to climate psychology articles) can also be used to assess journal performance. Based on this metric, *Climatic Change* stands out as the top journal with an h-index score of 217. It is followed by *Global Environmental Change* and the *International Journal of Environmental Research and Public Health*, which have h-index scores of 225 and 198, respectively, indicating strong performance in their contributions to the field.

**Table 3:** Most influential journals.

**Journals**	**Articles**	**Number of citations**	**Percentage of total articles**	**h-index**
Climatic change	28	617	2.09	217
Global environmental change	23	617	1.72	225
Sustainability Switzerland	22	476	1.64	169
International journal of environmental research and public health	20	359	1.49	198
Geoforum	20	675	1.49	141

### 3.5 Top articles

Information about the top articles is presented in Table 4. These articles are the ones with the highest number of citations across all fields in the Scopus database.

**Table 4:** The most influential articles are based on global citations.

**References**	**Number of citations**
Schlosberg (2013)	648
Newell and Mulvaney (2013)	536
Hickman et al. (2021)	412
Benjamin and Kumar (2017)	393
McCauley and Heffron (2018)	386

As observed, the article by McCauley and Heffron (2018) is the most cited work in the field of environmental justice, with 648 citations. This study explores how early research on environmental justice has expanded beyond traditional boundaries. It challenges the conventional understanding of “environment,” investigates the construction of injustice beyond mere inequality, and highlights the potential of pluralistic social justice concepts.

Another highly cited article, by Newell and Mulvaney (2013), with 536 citations, examines the political economy of the “just transition” to a low-carbon economy. The concept of a “just transition” has gained prominence in policy and political discourse, emphasizing the need to ensure that efforts to move toward a low-carbon future are equitable. This is particularly important for those currently lacking reliable energy access and living in energy poverty, as well as for those whose livelihoods depend on the fossil fuel industry. The study delves into the procedural and distributive aspects of energy policy and practice, especially concerning the just transition: ensuring energy access for those without it, achieving justice for workers in the fossil fuel sector, and managing potential conflicts that may arise from the simultaneous pursuit of energy and climate justice.

Hickman et al. (2021) examined climate anxiety among children and young people, specifically focusing on their views of government responses to CC. The study, which has been cited 412 times, reveals that CC is a widespread source of worry on a global scale, with 84% of respondents expressing at least a moderate level of concern. The data also show that these worries are often accompanied by various negative emotions, including sadness, anxiety, guilt, and helplessness. Additionally, more than 45% of participants reported that their climate-related emotions had a detrimental impact on their daily lives and functioning. Many respondents mentioned having frequent negative thoughts about CC. For example, 75% found the future to be frightening, and 83% believed that people have not taken proper care of the planet. The study also found that respondents viewed government actions to address CC negatively, feeling betrayed rather than supported. Furthermore, feelings of betrayal and distress related to CC were linked to a pessimistic view of government performance.

### 3.6 Scientific mapping

#### 3.6.1 Co-occurrence analysis of keywords

**Figure 5:**
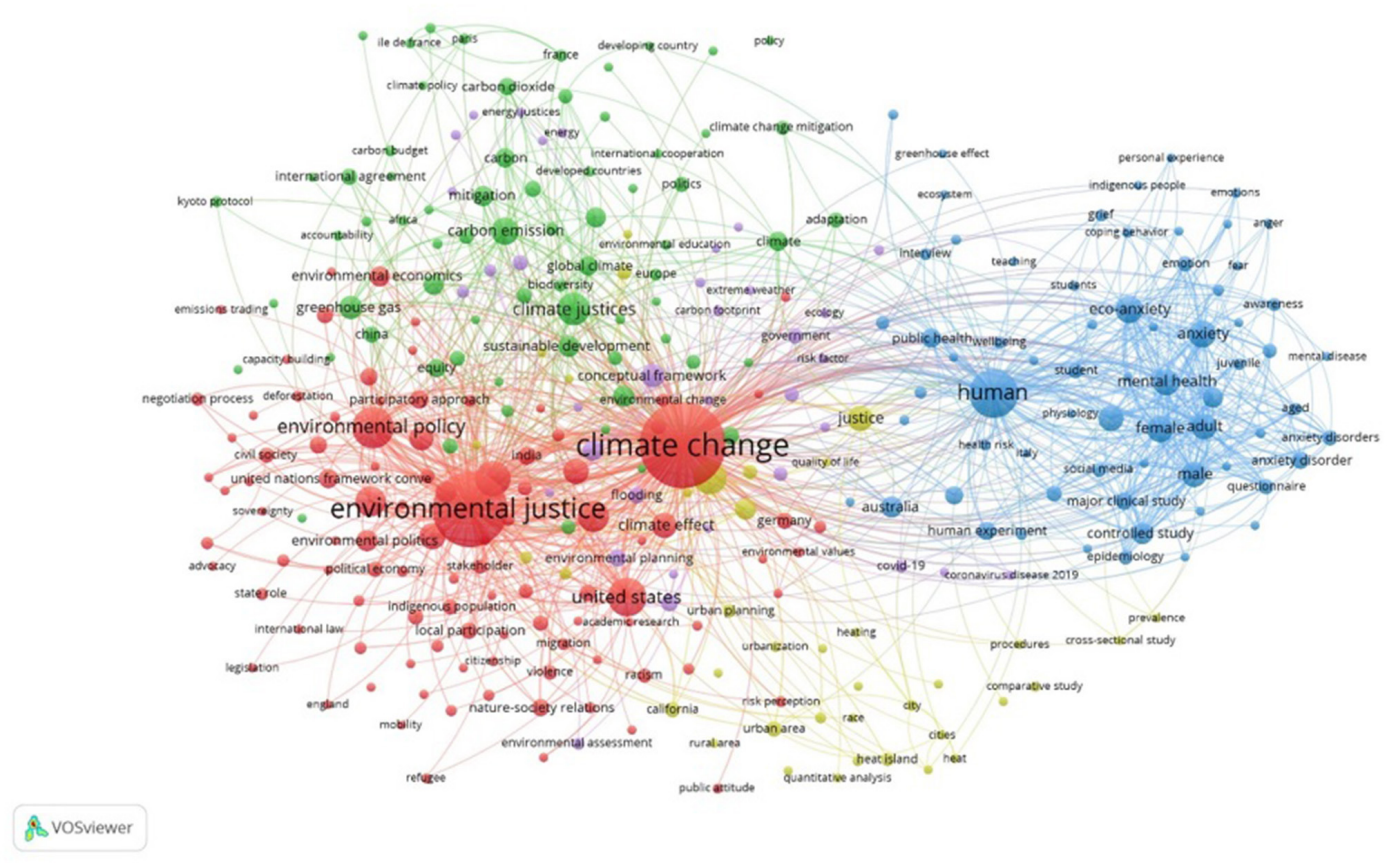
Keyword co-occurrence network.

Keywords can be seen as the fundamental content of published research, covering a variety of topics within a specific field. A cluster of interconnected keywords provides a visual display of the creation of scientific knowledge, showing connections, patterns, and the intellectual framework of the subjects being discussed (Lee and Su, 2010). Figure 5 illustrates a network of 313 keywords out of a total of 2,608. In this examination, a minimum of five occurrences was established as the threshold for including keywords.

The co-occurrence network is constructed by treating keywords as nodes, with their co-occurrence serving as the links between these nodes. Node size indicates the frequency of occurrence of each keyword in the dataset. Larger nodes represent more frequently used keywords. The frequency with which a pair of keywords appears together in a document is considered the weight of the link connecting them. Generally, co-occurrence refers to the simultaneous use of two keywords within a single document. Colors represent clusters of keywords that are closely related based on co-occurrence patterns, as determined by the software's clustering algorithm, typically using modularity-based techniques. The distance between nodes reflects the strength of co-occurrence: the closer two nodes are, the more frequently they co-occur in the same documents. Lines (edges) between nodes indicate co-occurrence links, with thicker lines representing stronger associations. The analysis of the keyword co-occurrence network revealed a thematic structure consisting of five clusters: the first and largest cluster comprises 102 subfields, the second cluster includes 69 subfields, the third contains 67, and the fourth and fifth clusters consist of 38 and 35 subfields, respectively.

The size of each keyword indicates its frequency in the articles. For example, terms like “climate justice,” “climate change,” and “eco-anxiety” are represented by larger nodes, showing their more frequent usage in the literature. Additionally, the proximity of keywords reflects how often they are used together in articles. In Figure 5, keywords that frequently co-occur are grouped into five distinct clusters, each represented by different colors. The first cluster comprises 117 keywords, with the highest link weight (3,104) associated with the keyword “climate change.” The second cluster includes 90 keywords, where “climate justice” holds the highest link weight (510). The third cluster consists of 70 keywords, with “human” having the highest link weight (1,634). The fourth cluster is made up of 36 keywords, with “social justice” having the highest link weight (589). Finally, the fifth cluster includes 35 keywords, with “conceptual framework” having the highest link weight (294).

**Figure 6:**
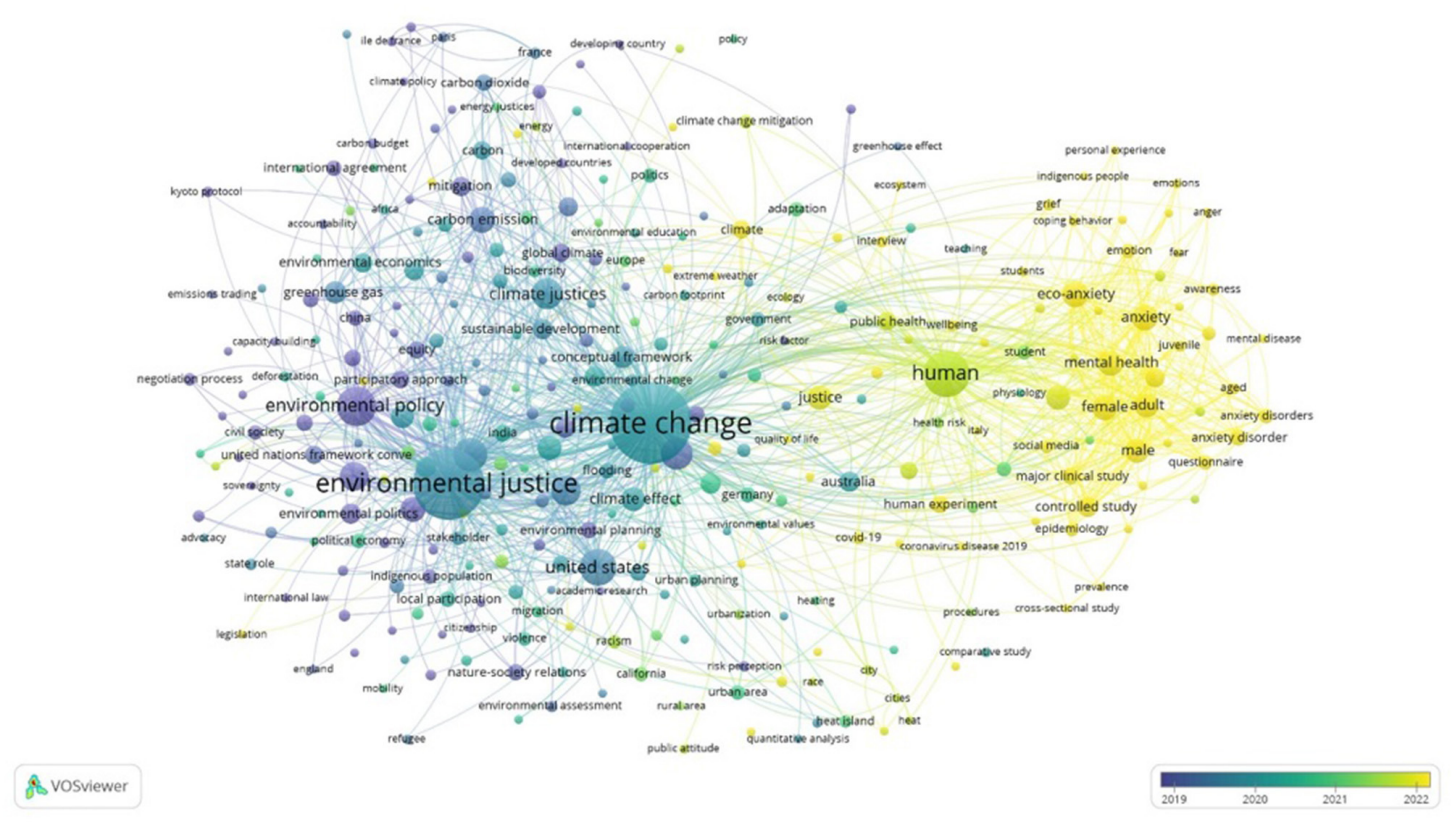
Layered map of the co-occurrence network.

Figure 6 displays the layered map of the keyword co-occurrence network. Similar to Figure 5, the size of each node represents the frequency of keyword usage. The color of each node indicates the average publication year, as shown by the color bar in the bottom right corner of the map. This bar reflects the publication years and trends in the field of CC and climate emotions.

Nodes that shift toward blue suggest that the keyword has been used in the literature for a longer period, indicating its longer historical presence in the discourse on CC and climate emotions. Conversely, nodes in yellow represent newer topics within this field. Table 5 lists the top 10 keywords with the highest link weights and co-occurrence.

**Table 5:** Keyword co-occurrence analysis top 10 keywords.

**Rank**	**Keyword**	**Occurrences**	**Total link strength**
1	Climate change	443	3,104
2	Environmental justice	330	2,083
3	Human	141	1,634
4	Environmental policy	105	768
5	United states	87	705
6	Female	41	651
7	Anxiety	47	637
8	Male	39	628
9	Eco-anxiety	44	622
10	Social justice	67	589

The ***co-occurrence network analysis*** of keywords revealed five main research clusters:

1. **CC cluster**: This cluster includes topics such as CC, environmental justice, environmental policy, adaptive management, and vulnerability. CC, a major challenge of the present century, impacts various aspects of human life. It leads to severe weather events like droughts, floods, and storms while affecting natural resources and ecosystems. Environmental justice, which focuses on the fair distribution of environmental benefits and burdens, is crucial, particularly for vulnerable communities in developing countries. Adaptive management, as a flexible approach, plays a key role in reducing community vulnerability to climate risks. Integrating these concepts can help find sustainable solutions to this global challenge.
2. **Climate psychology cluster**: This cluster includes terms such as human, mental health, ecological anxiety, public health, and pro-environmental behavior. The intricate connection between human emotions and the environment has attracted considerable interest. Ecological anxiety, fueled by worries about environmental decline and the planet's future, is having a growing impact on mental health, resulting in problems such as depression and insomnia. Conversely, engaging in pro-environmental actions, such as reducing consumption and recycling, can enhance mental health and foster a sense of control over one's life. It is essential to address environmental issues and encourage eco-friendly behaviors to improve public and mental health.
3. **Climate justice cluster**: This cluster includes terms such as climate justice, equity, carbon emissions, fossil fuels, biodiversity, and resilience. Climate justice and equity emphasize that the impacts of CC, particularly carbon emissions from fossil fuels, affect different communities unevenly, with vulnerable and minority groups suffering the most. While developed countries contribute more to global warming, developing countries often face the greatest harm. Biodiversity, essential for healthy ecosystems, is also threatened by CC and human activities. Resilience strategies are proposed to reduce community vulnerability. This cluster highlights the interconnectedness of environmental and social challenges and the need for a comprehensive, justice-based approach to achieve a sustainable future.
4. **Social justice cluster**: This cluster focuses on social justice, risk assessment, poverty, and equity in urban and rural areas. It addresses issues related to social inequality and efforts to achieve social justice in both urban and rural communities. Concepts such as risk assessment, poverty, and life equity are central to this cluster. Risk assessment helps identify and manage hazards, including poverty, which is a key factor in inequality. Life equity in urban and rural areas is a goal of sustainable development. Understanding differences in living standards can help develop strategies to bridge these gaps.
5. **Environmental effects cluster**: This cluster includes keywords such as environmental impacts, environmental planning, floods, droughts, and food security. These keywords highlight the close relationship between the environment, urban development, and food security. Natural events like floods and droughts, significantly influenced by CC, can have destructive effects on the environment, infrastructure, and food production. Effective environmental planning, such as identifying flood and drought-prone areas, designing drainage systems, creating green spaces, and managing water resources, can mitigate these impacts. Additionally, sustainable agriculture and crop diversification can enhance food security under unstable climate conditions.

#### 3.6.2 Thematic mapping through bibliographic coupling

**Figure 7:**
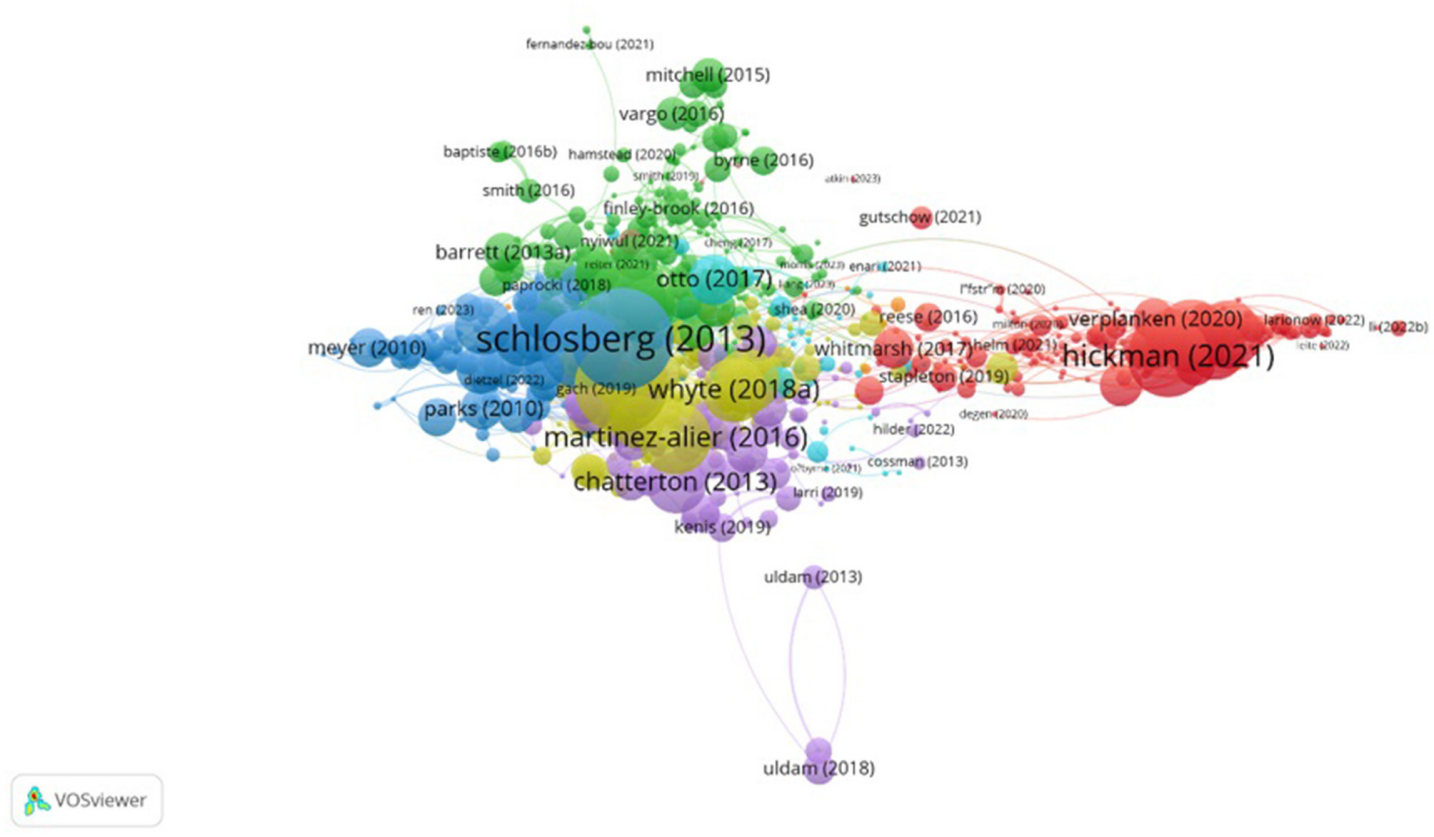
Bibliographic coupling.

Bibliographic coupling is used to understand the present intellectual structure of a field by grouping documents that cite similar references. This technique reflects the thematic proximity of recent research streams. Unlike citation-based rankings (e.g., global citation count in Section 3.5), this method reveals how recent publications are conceptually clustered based on shared bibliographic foundations. In this study, 163 documents met the minimum citation threshold and were grouped into six thematic clusters using VOSviewer. Each cluster corresponds to a specific focus area within climate-related psychological research. Out of a total of 1,333 initial documents analyzed through bibliographic coupling, 163 documents met the criterion of at least 21 citations. The most influential publications, based on citation power, are by Schlosberg (2013), with 648 citations, followed by McCauley and Heffron (2018) with 386 citations, and Sovacool (2016) with 393 citations. Table 6 displays the top 10 documents identified through bibliographic coupling analysis. Six distinct categories of documents emerged; illustrating key topics in the literature on the psychological impacts of CC (see Figure 7).

**Table 6:** The top 10 documents in bibliographic coupling and total link strength.

**Rank**	**Documents**	**Citation**	**Total link strength**
1	Pihkala, P. (2020a). Anxiety and the ecological crisis: an analysis of eco-anxiety and climate anxiety. *Sustainability* 12:7836. doi: 10.3390/su12197836	96	159.75
2	Pihkala, P. (2022). The process of eco-anxiety and ecological grief: a narrative review and a new proposal. *Sustainability* 14:16628. doi: 10.3390/su142416628	12	155
3	Pihkala, P. (2024). Ecological sorrow: types of grief and loss in ecological grief. *Sustainability* 16:849. doi: 10.3390/su16020849	0	137
4	Martinez-Alier, J., Anguelovski, I., Bond, P., Del Bene, D., Demaria, F., Gerber, J. F., et al. (2014). Between activism and science: grassroots concepts for sustainability coined by environmental justice organizations. *J. Polit. Ecol*. 21, 19–60. doi: 10.2458/v21i1.21124	175	111
5	Pihkala, P. (2022). Toward a taxonomy of climate emotions. *Front. Clim*. 3, 738154.	65	108
6	Pihkala, P. (2020). Eco-anxiety and environmental education. *Sustainability* 12(23), 10149.	228	106
7	Martinez-Alier, J., Temper, L., Del Bene, D., and Scheidel, A. (2016). Is there a global environmental justice movement? *J. Peasant Stud*. 43, 731–755. doi: 10.1080/03066150.2016.1141198	325	102
8	Henrique, K. P., and Tschakert, P. (2021). Pathways to urban transformation: from dispossession to climate justice. *Prog. Hum. Geogr*. 45, 1169–1191. doi: 10.1177/0309132520962856	26	96
9	Pihkala, P. (2024). Climate anxiety, maturational loss, and adversarial growth. *Psychoanal. Study Child*. 1-20.	1	76
10	Anguelovski, I., and Alier, J. M. (2014). The ‘Environmentalism of the Poor' revisited: territory and place in disconnected glocal struggles. *Ecol Econ*. 102, 167–176. doi: 10.1016/j.ecolecon.04005	125	76

The results of the bibliographic coupling analysis identified six separate clusters, each representing a specific thematic area. These clusters consist of papers with similar subject matter that are interconnected. To visually differentiate between clusters, papers within each group are color-coded. The categorization and explanation of each cluster will be provided in Table 7.

**Table 7:** Bibliographic coupling clusters related to psychological consequences of CC.

**Cluster**	**Cluster label**	**Number of publications**	**Representative publications**
1 (Red)	Psychological dimensions of eco-anxiety	212	Hickman et al., 2021; Baker et al., 2021; Clayton and Karazsia, 2020; Pihkala, 2020a,b; Clayton, 2020; Pihkala, 2018
2 (Green)	CC adaptation and climate justice	208	McCauley and Heffron, 2018; Schlosberg et al., 2017; Owen, 2020; Mercer et al., 2012; Popke et al., 2016
3 (Blue)	Environmental and social justice	203	Schlosberg, 2013; Bulkeley et al., 2014; Gössling et al., 2010; Bulkeley et al., 2013; Schlosberg, 2012
4 (Yellow)	Ecology of CC	162	Newell and Mulvaney, 2013; Martinez-Alier et al., 2014, 2016; Whyte K. P., 2018; Whyte K., 2018; Woodbury, 2019
5 (Purple)	Climate justice campaigns and movements	133	Chatterton et al., 2013; Routledge et al., 2018
6 (Turquoise)	Social vulnerability and environmental migration due to CCs	44	Otto et al., 2017; Ehrenfeucht and Nelson, 2023; Klepp and Herbeck, 2016; Milán-García et al., 2021

##### 3.6.2.1 Cluster 1: a comprehensive overview of ecological anxiety

**Cluster 1** provides a comprehensive view of ecological anxiety, extending beyond individual studies to depict a complex network of connections. This cluster primarily explores the resilience of children and youth in the face of climate adversities. Several studies emphasize the importance of resilience, particularly among children and young people, when confronting climate events (Baker et al., 2021; Clayton, 2020; Hickman et al., 2021). It is argued that parents and teachers should utilize resources to support environmental learning for their children in a way that enhances emotional wellbeing and hope.

Furthermore, climate anxiety and dissatisfaction with government responses among children and youth are widespread globally, impacting their daily functioning. Perceived failures by governments to address the climate crisis are associated with increased distress. Clayton and Karazsia (2020) focused on the development and validation of measures for climate anxiety, finding that climate anxiety, particularly among young adults, is not uncommon. This concern can be distinguished from more severe impacts on an individual's life. Climate anxiety is associated with emotional responses rather than behavioral reactions to CC. Clayton (2020) noted that CC affects mental health, with harmful effects on physical health, mental wellbeing, and social relationships resulting from exposure to extreme weather events related to CC. Recently, there has been a growing interest in the potential impacts of CC on mental health, particularly through emotional responses such as increased anxiety. This study examines the nature of climate anxiety, provides evidence of its existence, and discusses coping strategies. Climate anxiety seems to be a real issue that requires clinical attention. It is important to distinguish between adaptive and maladaptive levels of this anxiety. Emphasis on individual mental health should not divert attention from the necessary social responses to address CC. Pihkala (2018) suggested that framing a situation merely as a threat or opportunity is insufficient. Religious communities and methods that incorporate spirituality play a significant role in empowering individuals to process profound feelings and existential questions.

**Cluster 2** focuses on the relationship between CC, adaptation, and environmental justice. This cluster highlights the critical role of adaptation as a significant factor in maintaining resilience. McCauley and Heffron (2018) introduced the concept of a just transition, emphasizing the need to address the new realities of ongoing CC s. The impacts are no longer confined to job losses in a few developed countries but affect communities globally, including both the Global North and the Global South. The effects extend beyond society to reshape our environment, global ecosystems, and future climates. They also identified restorative justice as a crucial dimension that needs further development, as procedural justice alone may not sufficiently ensure that offenders are held accountable and victims receive redress. Schlosberg et al. (2017) examined just policy and transformation, noting that despite a discursive divide between government focus on risk-based or resilience approaches and community concerns about basic needs and daily living capabilities, consultative participation in adaptation planning can address justice issues and demonstrate transformative functions. Popke et al. (2016) argue that increased attention to vulnerability in climate policy has the potential to advance environmental justice goals in the region. Mercer et al. (2012) explored farmer adaptation to CC and climate justice, finding that CC impacts agricultural production in areas where indigenous varieties are maintained. They proposed a transgenic adaptation strategy to address CC impacts, suggesting that agroecological and evolutionary approaches offer an alternative adaptive strategy for smallholder agriculture.

**Cluster 3** emphasizes the importance of environmental and social justice. Schlosberg (2013) theorizes about environmental and social justice, suggesting that the spatial expansion of this concept includes a wider range of issues horizontally, a deeper analysis of global environmental injustices vertically, and a conceptual exploration of the human-nonhuman relationship. The study argues that recent extensions of the environmental justice framework have shifted the conversation to a new realm where the environment and nature are viewed as essential in creating conditions for social justice. Gössling et al. (2010) discussed the future adaptation of tourism and climate policy. They concluded that given the observed and projected growth rates in emissions, technology and management alone will not be sufficient to achieve even modest reductions in greenhouse gas emissions in this sector. They emphasize the crucial role of social and behavioral changes in achieving climate-sustainable tourism and note that fundamental changes are necessary to align the increasing global demand for holidays and business travel with international climate policy goals, particularly to limit anthropogenic global warming to <2°C.

**Cluster 4** focuses on indigenous perspectives in addressing CC. It provides a comprehensive and dynamic understanding of ecological approaches and local viewpoints to address environmental injustices and tackle climate trauma. Newell and Mulvaney (2013) examine the political economy of a “just transition” to a low-carbon economy. The concept of “just transition” is increasingly prominent in policy and political discourse, emphasizing the need to ensure that efforts to guide society toward a lower-carbon future are supported with considerations of equality and justice for those currently lacking reliable access. Martinez-Alier et al. (2014, 2016) highlight the rising number of ecological distribution conflicts globally, reflecting the existence of a global rural and urban movement for environmental justice. This movement has generated a wide range of concepts and slogans to characterize and engage with these conflicts. These concepts include environmental racism, public epidemiology, environmentalism of the poor and Indigenous peoples, biopiracy, environmental debt, climate justice, food sovereignty, land grabbing, and water justice, arising from social and environmental activism. Whyte K. (2018) discusses how some Indigenous perspectives on CC can perceive the present as a predetermined dystopia. Engaging in intergenerational dialogues with both children and ancestors can help shape local responses to CC, offering an alternative to the fear-driven approach often associated with an impending crisis. Woodbury (2019) considers CC as a psychological-social defense mechanism against the catastrophic impacts of climate science. From a traumatology perspective, the climate crisis creates a new, unprecedented, and profound type of trauma known as “climate trauma.” This trauma represents a persistent and growing threat to the biosphere and collective human identity. The lack of response to the climate crisis is attributed to climate trauma, and solving this crisis requires fundamental changes in social and cultural approaches.

**Cluster 5** focuses on just transition and climate justice campaigns and movements. Newell and Mulvaney (2013) analyze the political and economic challenges associated with transitioning to a low-carbon economy in a fair and equitable manner. A just transition aims to ensure fairness and equality during this shift, particularly for those without access to energy, living in energy poverty, or reliant on fossil fuels for their livelihoods. Chatterton et al. (2013) discovered that climate justice inspires the creation of political movements that manifest through collective actions, fostering solidarity among different struggles. These alliances have the potential to reshape the conversation surrounding CC. Emphasizing these principles can underscore the significance of climate justice in political strategies and performance. The article concludes by examining various interpretations of climate justice and the conflicts that arise in emerging climate policy frameworks.

**Cluster 6** emphasizes social vulnerability and environmental migration due to CC. Otto et al. (2017) explores social vulnerabilities related to CC, aiming to identify social and demographic groups at higher risk across four dimensions of wellbeing (health, safety, food security, and displacement) in a range of geographical locations. The authors conclude that CC will exacerbate existing vulnerabilities and inequalities, raising concerns about climate justice, particularly in intergenerational dimensions. The research also indicates limited evidence of critical thresholds, meaning it is unclear when CC will become severe enough to cause a sudden and significant increase in social vulnerability. Ultimately, it underscores the need for attention to climate justice and appropriate measures to support vulnerable groups against CC. Ehrenfeucht and Nelson (2023) highlight that CC increasingly impacts development patterns and where people live. Climate adaptation policies should assist residents in decisions about staying, relocating, or choosing new places to live. Klepp and Herbeck (2016) point out a global legal impasse concerning environmental migrants. Milán-García et al. (2021) discovered a normative shift in the discourse, transitioning away from ideas like vulnerability and security toward more justice-oriented concepts like climate justice and human rights. Future research in this area should compare findings from human-centered research with those focusing on other living beings (Table 7).

## 4 Discussion

In this study, a bibliometric analysis was conducted to provide an overview of the existing literature on climate emotions. By identifying crucial articles, authors, journals, and advancements in this area, this research offers a valuable foundation for policymakers, researchers, and educators interested in exploring this subject. Furthermore, by recognizing different communities—including (1) foundational knowledge bases, (2) current research frontiers, and (3) future directions for the field—this study elucidates essential areas of focus and pinpoints knowledge gaps that require further exploration. The study first examined publication trends, revealing a significant growth in research within this field. Notably, in the past three years, 740 articles, representing 55.1% of all published works, were released. This surge highlights an increasing focus on CC and its related emotions, a trend expected to continue. The highest volume of articles on CC and associated emotions was in 2023, with 331 papers, while the lowest was in 2010, with only 12 papers. Since 2019, there has been a notable increase in scientific output in this area, particularly with the emergence of climate justice and environmental justice themes, a trend that continued through 2023. Keywords such as “ecological anxiety” and “mental health” reflect a growing focus on these issues in recent years.

The analysis of the most prolific authors in the field reveals that B. Sovacool from Denmark and P. Bond from South Africa are the top contributors, with 597 (59.7%) and 186 (13.9%) citations, respectively. I. Anguelovski ranks third with 122 publications (9.2%). This study also identifies the countries and institutions that have produced the most articles in this field, which can serve as a benchmark for identifying leading universities in CC and related research. The findings indicate that the United States leads in most areas, holding the highest position for article production on this topic. This suggests that U.S. institutions are at the forefront in this field and could be ideal destinations for researchers. Following the U.S., the United Kingdom, Australia, Canada, and Germany are the top five countries producing research in this area. Among the top 10 countries, India is the only representative from Asia, ranking 10th with 36 articles. Additionally, the collaboration network among countries in this field, as indicated by the Scopus database, shows that the United States, the United Kingdom, Canada, and Australia have the most extensive research collaborations.

The analysis of publications in the field of CC and climate emotions reveals that articles are published in 60 journals indexed in Scopus. Among these, “Climatic Change” leads with 28 articles (2.09%), focusing primarily on climate variability and change, its causes, consequences, and interactions. “Global Environmental Change” ranks second with 23 articles (1.72%), and “Sustainability Switzerland” ranks third with 22 articles (1.64%). These journals are interdisciplinary and cover a broad range of topics related to CC. During citation analyses, several highly cited articles such as McCauley and Heffron (2018) and Newell and Mulvaney (2013) emerged. These articles do not primarily focus on psychological constructs but rather on environmental and energy justice. Their presence in the citation network highlights the interdisciplinary nature of CC discourse and demonstrates how systemic and justice-related issues may indirectly impact psychological outcomes such as eco-anxiety, climate grief, and perceived agency. It is important to note that these articles do not explicitly examine individual psychological responses to CC. Their inclusion in the analysis reflects broader thematic overlaps rather than direct psychological relevance.

### 4.1 Bibliographic coupling clusters

This cross-mapping illustrates how conceptual themes identified through content-based bibliographic coupling are reflected in the structural patterns of keyword co-occurrence (see Table 8). It strengthens the interpretive framework of the study by showing the complementarity of the two analytical approaches.

**Table 8:** Cross-mapping of thematic and keyword clusters.

**Thematic cluster (Bibliographic coupling)**	**Dominant keywords (Co-occurrence analysis)**	**Description of the link**
Ecological anxiety and psychological impacts (Cluster 1)	Eco-anxiety, anxiety, mental health, youth	Focus on psychological responses to CC, particularly among youth and vulnerable groups
Climate justice and just transition (Clusters 2 and 5)	Climate justice, fossil fuels, equity, resilience	Overlaps with structural and distributive justice, and climate-related social movements
Social and environmental justice (Cluster 3)	Social justice, environmental policy, inequality	Convergence of climate governance and justice-oriented frameworks
Indigenous and spiritual perspectives (Cluster 4)	(Less explicit in keywords; indirectly reflected in: human, climate trauma)	Emphasizes traditional knowledge, existential grief, and spiritual coping in climate discourse
Social vulnerability and environmental migration (Cluster 6)	Vulnerability, migration, poverty, food security	Clear alignment with terms reflecting climate-induced displacement and socio-economic vulnerability

### 4.2 Implications of findings

Since 2019, literature on climate emotions has rapidly expanded, underscoring its emerging importance as a critical scientific domain. The increase in publications since 2020, characterized by a rise in both article volume and journal diversity, indicates that this year was a pivotal moment for the development of this literature. Research on climate emotions is primarily influenced by the intersecting fields of psychology, education, and environmental studies, with support from leading researchers and key works falling within this scope. Our findings not only highlight the relevance of these domains in current contexts but also help identify potential gaps in the literature. Researchers from other disciplines, such as the life sciences, medicine, humanities, etc., may find this information useful for recognizing broader educational trends and addressing specific needs related to the impact of CC on emotions within their respective fields.

We also found that most research in this area has been predominantly concentrated in three countries: the United States, the United Kingdom, and Australia. This finding aligns with Parsons and Crease (2024), which indicates that a significant portion of climate justice research is centered on the United States. However, since CC and its consequences have global dimensions, future researchers should place greater emphasis on international collaboration and the needs of developing countries. Advances made in these three industrialized nations may serve as valuable lessons for other countries with varying levels of research and operational development.

One notable observation from this study is the emergence of new concurrent keywords, such as “Coping behavior” and “Juvenile,” around 2022, highlighting the evolving landscape of climate emotion research. Additionally, recent studies indicate a stronger focus on diverse groups, such as women, men, youth, and adults, as well as the effects that climate emotions and mental health might have on individual behaviors, particularly climate-related behaviors among different demographic groups.

A cluster of documents has emerged, illustrating how communities have prepared for and responded to the impacts of CC. Close acknowledgment and tracking of advances in psychology within the context of CC, as seen in this cluster, is essential. It is therefore unsurprising that publications in this cluster are newer compared to others. Ultimately, our analyses reveal a unique collection of documents related to climate psychology, highlighting the application of psychological concepts to systematically develop mental processes aimed at problem-solving. Psychological processes needed to explain individuals' emotions have garnered significant attention over the past few decades and have emerged as a novel aspect within syntheses and reviews. The co-occurrence analysis provides a practical structural framework for future researchers entering this field.

### 4.3 Addressing emerging concepts in climate justice literature

As the field evolves, so does its terminology and conceptual scope. While the current study captures dominant themes such as “climate anxiety,” “eco-anxiety,” and “environmental justice,” it is also important to reflect on emerging climate justice discourses that are shaping recent scholarship. Concepts such as transformative adaptation, intergenerational equity, procedural climate justice, and indigenous epistemologies have gained increasing traction, particularly in publications post-2022. To enhance conceptual currency, future bibliometric studies should integrate these emerging frameworks into keyword selection and clustering logic. For example, “loss and damage,” “just transition,” and “climate trauma” now serve as not only research foci but also rallying terms in both scientific and policy discourses. Moreover, climate justice is becoming intersectional, extending into gender, race, and youth-related domains. To reflect this trend, we have expanded our keyword analysis to include terms such as “youth resilience,” “climate adaptation policy,” and “transformational justice,” which emerged in publications from 2022 onward. This step ensures alignment with the cutting-edge vocabulary and theoretical underpinnings of the climate justice discourse.

### 4.4 Current situation and outlook

Our bibliographic coupling analysis sheds light on the current landscape of literature on climate emotions. We have identified distinct clusters that showcase the application of environmental psychology in the context of CC. These clusters collectively indicate the emergence of a new field known as climate psychology, which uses psychological research to improve learning, education, and adaptation strategies to mitigate the adverse effects of CC. Additionally, we have noticed that two specific domains within the climate emotions literature, epidemiology and social sciences, have evolved independently. These findings offer an opportunity for integrating knowledge across domains and serve as valuable starting points for researchers in related fields looking to engage with this literature.

By using co-word analysis, we can predict the developmental path of this field. The primary clusters, which include CC, environmental justice, and the connection between humans, indicate that this area is evolving naturally by incorporating themes from past and present discussions. The emergence of the “human” cluster suggests recent progress in discussing factors and aspects related to human characteristics. CC, viewed through the lens of climate psychology and its associated social variables, has significantly improved our understanding of the new challenges posed by CC. Climate psychology explores the impact of CC and environmental issues on mental health and human behavior, examining how awareness of CC can affect emotions, attitudes, and individual and collective behaviors. The appearance of these concepts as key terms implies that research on CC should consider these psychological and social impacts. The co-word analysis highlights clusters of keywords, such as “climate anxiety” and “environmental justice,” that reflect both CC-specific and broader environmental concerns. While this study focuses on the psychological impacts of CC, the frequent co-occurrence of terms like “eco-anxiety” and “environmental degradation” suggests that these domains are interconnected in the literature. This overlap underscores the need for future research to differentiate the psychological effects of CC from those of other environmental stressors, potentially through targeted empirical studies or systematic reviews that isolate these variables. In future studies, it would be beneficial to combine bibliometric methods with systematic content analysis to validate and enhance our findings. This approach would enable a more thorough evaluation of study designs, interventions, and outcomes, thereby overcoming limitations associated with the interpretive depth of bibliometric techniques.

### 4.5 The limitations

This study's reliance on Scopus presents certain limitations, including the potential exclusion of regionally or linguistically diverse publications not indexed by Scopus. Comparative studies have shown substantial variance in database coverage depending on the field of inquiry (Gusenbauer and Haddaway, 2020; Zhu and Liu, 2020). Therefore, while Scopus offers reliable metadata and citation tracking features, the findings should be interpreted with an understanding of this constraint. As Gusenbauer (2022), Gusenbauer and Haddaway (2020), and Zhu and Liu (2020) emphasize, no single bibliographic database can fully encompass the diversity of scholarly output across all fields. Future studies would benefit from integrating data from multiple sources such as Web of Science, Dimensions, and Google Scholar to enhance coverage and reduce database-specific bias.

## 5 Conclusion

Using bibliometric analysis, we have mapped the intellectual structure of the literature on CC and its emotional consequences. We have identified key publications, researchers, and journals that contribute to the topic and have examined the relationships among them. We have observed that the literature in the past 4 years has significantly expanded compared to 2010, indicating the emerging nature of this field. This area is primarily led by psychologists, education researchers, and environmental scientists, with most research concentrated in the social sciences. However, we have noticed that only a limited number of scholars consistently engage with this subject. By highlighting the communities that are emerging within the existing literature, we have identified the dominant disciplines such as life sciences and medicine, and topics like epidemiology and climate psychology. We have also uncovered significant gaps in this field related to disciplines and topics that we had expected to see as distinct communities. Looking ahead to the future of the field, we have observed that emotions and human characteristics are becoming increasingly important in the context of CC. Given the impact of CC on policy agendas, our analyses provide valuable insights and an overview of the current literature landscape.

Given the interdisciplinary nature of climate emotions, it is essential to create more opportunities for collaboration across different fields to enrich research studies. Examining the topic of climate emotions among various sectors and communities can offer valuable insights and help identify both differences and commonalities that might be overlooked in studies confined to a single discipline. Additionally, conducting similar research in other citation databases, such as Web of Science, would allow for broader comparisons and validation of findings. This would contribute to a more well-rounded body of knowledge and help shape more effective and inclusive policies and interventions to address climate-related emotional responses. These efforts would not only enhance the current research landscape but also ensure that future studies are better equipped to address the complex challenges posed by CC.
